# Metronidazole Appears Not to Be a Human Teratogen: Review of Literature

**DOI:** 10.1155/S1064744997000574

**Published:** 1997

**Authors:** Barbara J. Struthers

**Affiliations:** G.D. Searle & Co. 5200 Old Orchard Road Skokie IL 60077 USA

## Abstract

Metronidazole is used to treat trichomoniasis, bacterial vaginosis, and other diseases. As is the case with many drugs, physicians often hesitate to use it during pregnancy, particularly in the first trimester. A review of the nearly four decades' worth of published literature on metronidazole use in pregnant women indicates that it is not teratogenic, regardless of the trimester in which it is used. On the other hand, a number of published studies indicate that bacterial vaginosis and trichomoniasis are associated with preterm birth and low birth weight. Treatment of these conditions with metronidazole during pregnancy may decrease the incidence of preterm birth and low birth weight, thus potentially decreasing the complications that can result from prematurity.


Infectious Diseases in Obstetrics and Gynecology 5:326–335 (1997)
© 1998 Wiley-Liss, Inc.

### Metronidazole Appears Not to Be a Human Teratogen: Review of Literature

**Barbara J. Struthers**

*G.D. Searle & Co., Skokie, IL*

ABSTRACT

**Metronidazole is used to treat trichomoniasis, bacterial vaginosis, and other diseases. As is the case with many drugs, physicians often hesitate to use it during pregnancy, particularly in the first trimester. A review of the nearly four decades' worth of published literature on metronidazole use in pregnant women indicates that it is not teratogenic, regardless of the trimester in which it is used. On the other hand, a number of published studies indicate that bacterial vaginosis and trichomoniasis are associated with preterm birth and low birth weight. Treatment of these conditions with metronidazole during pregnancy may decrease the incidence of preterm birth and low birth weight, thus potentially decreasing the complications that can result from prematurity. Infect. Dis. Obstet. Gynecol. 5:326–335, 1997.** © 1998 Wiley-Liss, Inc.

KEY WORDS
metronidazole; teratogenicity; pregnancy; premature rupture of membranes; preterm birth; trichomoniasis; bacterial vaginosis

Metronidazole, a nitroimidazole that has been on the U.S. market since 1961, is used to treat trichomoniasis, bacterial vaginosis (BV), amebiasis, giardiasis, and numerous anaerobic bacterial infections. It has been shown to cause cancer in mice and rats (but not in other species) and to be mutagenic in some bacterial systems and even embryotoxic in some in vitro tests.[1,2] The relevance of these studies to humans is questionable. This paper reviews available human data and some animal studies that examine the potential for teratogenicity in humans.

### HUMAN STUDIES

In a study of women undergoing therapeutic abortion, Heisterberg[3] showed that metronidazole crossed the placental membrane; placental concentrations were approximately 50–65% of those in maternal plasma. Amon and Amon[4] found that, on occasion, the fetal metronidazole tissue concentrations exceeded those found in maternal plasma. No controlled clinical trials have been conducted specifically to determine whether metronidazole is teratogenic in humans. However, numerous reports have been published in which the effects of metronidazole taken to treat an infection during pregnancy were examined. Tables 1–3 give a synopsis of findings in pregnant women. The larger studies are briefly summarized below.

Rosa et al.[5] used a large health management organization's database including 104,339 women who gave birth to normal children and 6,564 women who delivered babies with anomalies. These included 1,083 women treated with metronidazole in the first trimester. Of these babies, 1,020 were normal and 63 were born with some anomaly; the relative risk of an anomaly being associated with metronidazole treatment in this cohort was 0.92. These authors reported an increased risk of spontaneous abortion in patients prescribed metronidazole in the 120-day period prior to spon-

Correspondence to: Dr. Barbara J. Struthers, G.D. Searle & Co., 5200 Old Orchard Road, Skokie, IL 60077.

Received 26 September 1997
Accepted 15 December 1997
Review Article

TABLE 1. Number of congenital anomalies reported in studies in which metronidazole was used during pregnancy

| Author | Mothers, n (control) | Infants, n[a] (control) | Trimester treated, n[b] | | | Defects, n (control) | Stillbirth/abortus/death, n (control) |
|---|---|---|---|---|---|---|---|
| | | | I | II | III | | |
| | | | Controlled studies | | | | |
| Rosa, et al [5] | 1083 | 1083 | 1083 | | | 63 | 135 |
| | | (104,339) | | | | (6564) | (not stated) |
| Morgan [6] | 597 | 605 | 62 | 284 | 251 | 12 | 3 |
| | (9757) | (9757) | | | | (172) | (not stated) |
| Heinonen, et al [7] | 31 | 31 | 31 | | | 4 | 0 |
| | (50282) | (50282) | | | | (3248) | (not stated) |
| Piper, et al [8] | 1307 | 1318 | 1318 | | | 96 | 4 |
| | (1311) | (1320) | | | | (80) | (8) |
| Hauth, et al [9] | 426 | 426 | | 426 | | 0 | 0 |
| | (190) | | | | | (0) | (0) |
| Morales [10] | 44 | 44 | | 44 | | 0 | 0 |
| | (36) | (36) | | | | (0) | (0) |
| Robinson, Mirchandi [22] | 277 | 281 | 14 | 87 | 176 | 6 | 1 |
| | (277) | (277) | | | | 7 | (not stated) |
| | | | Uncontrolled studies | | | | |
| Scott-Gray [11] | 295 | 295 | 79 | 113 | 103 | 5 | 10 incl. 2 TAB |
| Peterson, et al [12] | 206 | 212 | 55[c] | 78[c] | 84[c] | 8 | 13 |
| Magnin, et al [13] | 58 | 58 | 3 | 23 | 32 | 0 | 0 |
| Perl [14] | 151 | 151 | 4 | 77 | 70 | 3 | 3 |
| Pagnozzi, et al [15] | 61 | 61 | 25 | | | 0 | 0 |
| Sands [16] | 123 | 124 | | | | 1, familial | 2 |
| Watt, Jennison [17] | 19 | 19 | | | | 1 | 1 + 1 TAB |
| Zacharias, et al [18] | 34 | 34 | | | | 0 | 1, Rh factor |
| Williams [19] | 14 | 14 | | | | 0 | 0 |
| Parker, et al [20] | 36 | 36 | | | | 0 | 0 |
| Kotcher, et al [21] | 34 | 34 | | | 34 | 1 | 0 |
| Nigam, et al [24] | 7 | 7 | | | | 0 | 3 |
| Beard [25] | 23 | 23 | 23 | | | 5 | 3 |
| Scott-Gray [26] | 40 | 40 | 13 | 15 | 12 | 0 | 0 |
| Schram, Kleinman [27] | 26 | 26 | | | | 0 | 1 |
| Lyon, et al [28] | 9 | 9 | 1 | 4 | 4 | 0 | 0 |
| Andrews, Andrews [29] | 19 | 19 | 7 | | | 0 | 2 |
| Cantú, Garcia-Cruz [30] | 2 | 2 | 2 | | | 2 case reports | |
| Pereyra, Lansing [31] | 42 | 42 | | | | 0 | 0 |
| Glowinski, Chrusciel [32] | 11 | 11 | 2 | 0 | 9 | 0 | 2 |
| Gardner, Dukes [33] | 18 | 18 | | | | 0 | 0 |
| Whitelaw, et al [34] | 5 | 5 | 1 | | | 0 | 0 |
| Rodin, Hass [35] | 61 | 61 | 13 | 29 | 19 | 0 | 4 |
| Csonka [36] | 7 | 7 | | | | 0 | 0 |
| Monroe [37] | 25 | 25 | 10 | 5 | 10 | 0 | 0 |
| Ross, van Middelkoop [39] | 99 | 99 | | | | 0 | 0 |
| Carvajal [40] | 1 | 1 | | 1 | | 1 case report | |
| Perl, Ragazzoni [41] | 49 | 49 | | | | 0 | 0 |
| Robinson, Johnston [42] | 92 | 93 | | | | 3 | 0 |
| Doyle, Rolfe [43] | 1 | 1 | | | 1 | 0 | 0 |

TAB: therapeutic abortion.
[a]Liveborn.
[b]Trimester treated, if stated by author. Blanks indicate that trimester treated is not given.
[c]Multiple courses of therapy.
Blank spaces indicate that the data were not reported.

taneous abortion compared with those prescribed metronidazole 300–180 days prior to delivery; no excess risk was found when the population was compared with women who underwent elective abortion. The authors state that metronidazole therapy is frequently avoided during pregnancy; this may reflect no avoidance of therapy prior to elective abortions, compared with avoidance of

TABLE 2. Congenital anomalies reported in published studies of metronidazole treatment in pregnancy

| Author | Metronidazole* | Controls |
|---|---|---|
| | Controlled studies | |
| Morgan [6] | 7 major, 5 minor (2%) | 96 major, 76 minor (1.8%) |
| | Accessory digit (I) | |
| | Anencephaly + meningomyelocele (I) | |
| | Heart valve atresia + situs inversus (II) | |
| | Cleft palate + harelip (II) | |
| | Myotonia congenital (familial) (II) | |
| | Down's syndrome (III) | |
| | Fallot's tetralogy (III) | |
| | Atrial septal defect + accessory digits (III) | |
| | Intestinal obstruction (banding) (III) | |
| | 2-Single unbilical artery (II, III) | |
| Piper [8] | 96 total defects | 80 total defects |
| | 16 CNS | 19 CNS |
| | 6 heart | 8 heart |
| | 6 GI | 5 GI |
| | 35 musculoskeletal | 29 musculoskeletal |
| | 38 urogenital | 25 urogenital |
| | 2 respiratory | 2 respiratory |
| | 7 multiple organ systems | 2 chromosomal |
| | | 6 multiple organ systems |
| Robinson [22] | Hypospadias (II) | Harelip |
| | Skin tag, ear (II) | Congenital heart disease |
| | Hydrocephalus (III) | Spina bifida |
| | Club foot (III) | Blood dyscrasia |
| | Harelip and cleft palate (III) | Anencephalus |
| | Congenital hip (III) | 2 club foot |
| | Uncontrolled studies | |
| Peterson [12] | Cyanotic heart disease (II) | |
| | Hydrocephaly (III) | |
| | Calcaneus valgus (I, II) | |
| | Hypospadius (I, II) | |
| | Pyloric stenosis (II) | |
| | Hydrocele (I) | |
| | Syndactyly (I) | |
| | Small testicle (III) | |
| Perl [14] | Down's syndrome (II) | |
| | Cardiac defect (II) | |
| | Hemangioma | |
| | Anencephaly (stillborn) | |
| Beard [25] | 2-Hydrocele | |
| | Congenital hip dislocation | |
| | Unilateral metatarsus varus | |
| | Mental retardation (parents also) | |
| Robinson, Johnston [42] | Umbilical hernia | |
| | Hydrocephalus | |
| | Small skin tags on ear | |
| Scott-Gray [11] | Microcephaly + meningocele | |
| | Multiple defects | |
| | Cardiac lesion | |
| | Mild hydrocephaly | |
| Watt, Jennison [17] | Anencephaly | |
| Sands [16] | Polydactyly (familial) | |
| Kotcher [21] | Crooked foot | |
| Zacharias [18] | Rh factor (stillborn) | |
| Cantú [30] | Midline facial defects (I) | |

*Roman numeral following defect indicates trimester treated, when reported.

TABLE 3. Summary of published data reviewing metronidazole treatment in pregnancy

| Mothers | Infants | Defects | % infants with congenital anomalies |
|---|---|---|---|
| Total number treated with metronidazole in controlled studies | | | |
| 3,765 | 3,788 | 181 | 4.77% |
| Total number of controls | | | |
| 166,192 | 166,201 | 10,071 | 6.06% |

Total number treated with metronidazole, all studies:

| Mothers, n | Infants, n | Trimester treated, n* I | II | III | Major & minor defects, n | Stillbirth/abortus/death, n |
|---|---|---|---|---|---|---|
| 5,333 | 5,369 | 2,667 | 1,186 | 805 | 211 (3.93%) | 189 (3.52%) |

*When known.

therapy in those continuing pregnancy. The manner of presentation of abortion data makes recalculation impossible. The authors have recognized that their results are difficult to interpret, since only computer records of prescription dates, delivery, and abortion, but no medical details, were available, and stated that no conclusions can be drawn.

Morgan[6] studied 880 pregnant women: 283 were untreated, and the remaining 597 received 7–10 days of oral metronidazole therapy, 200 mg, three times daily. Sixty-two were treated in the first trimester, 284 in the second trimester, and 251 in the third trimester. The incidence of small-for-dates births and premature births were not altered. Major congenital anomalies were found in 1.7% of untreated patients and 1.2% of treated patients. In untreated patients, 2.7% of births had some congenital anomaly, compared with 2% in the metronidazole group. The incidence of congenital anomalies among 9,757 births occurring in the same hospital during that period was 1.8%.

Heinonen et al.[7] examined malformations in relation to exposure to various drugs in 50,282 mother-child pairs, including 31 exposed to metronidazole. There were four malformed children born to metronidazole-treated mothers, and a standardized relative risk estimate of 2.02, which was not significant. Two of these exposures occurred during the first trimester, giving a relative risk of 1.39, which was also not significant.

Piper et al.[8] compared two cohorts of pregnant women from the Tennessee Medicaid enrollment files. The exposed cohort included 1,307 women who received a metronidazole prescription between 30 days before and 120 days after their last normal menstrual period; the controls were 1,311 comparable women who had not received metronidazole. Results were comparable for the two groups in terms of twin gestations, fetal viability at delivery, gender ratio, very low birth weight, and major birth defects. The adjusted risk ratio for all birth defects among metronidazole-exposed infants compared with controls was 1.2 (0.9–1.6). The specific types of defects in exposed and unexposed groups are similar and are listed for both groups in Table 2.

A group of 624 women with a history of preterm birth were enrolled in a metronidazole treatment trial during their second trimester of pregnancy.[9] The treatment group included 433 women, while the control group included 191 women who received a placebo. The treatment group received metronidazole, 250 mg, three times daily for seven days, plus erythromycin, 333 mg, three times daily for 14 days. The incidence of preterm delivery (birth at or before 37 weeks' gestation) was significantly decreased only among the 263 women who had BV when they entered the study: 31% of the 176 treated women with BV delivered preterm, compared with 49% of the 87 untreated women with BV ($P$=0.006). No congenital anomalies were identified in either the treatment or the placebo group. (Personal communication to the author from Dr. Hauth.)

Morales et al.[10] enrolled in a clinical trial 80 women with a history of preterm birth who had BV; congenital anomalies were ruled out at study entry by ultrasonography. Forty-four patients received treatment with metronidazole, 250 mg, three times daily for seven days, and the placebo group received identical-appearing vitamin C tablets. More than one admission to the hospital for preterm labor was experienced by 78% of women in the placebo group, compared with 27% of treated patients. Delivery at <37 weeks occurred in 18% of treated patients and 39% of patients in the placebo group ($P<0.05$). No congenital anomalies were identified in either group.

Scott-Gray[11] monitored 295 pregnant women who were treated with metronidazole; 79 were treated during the first trimester, 113 in the second, and 103 in the third. Five infants were born with defects (see Tables 1 and 2).

Peterson[12] reported on 212 infants born to 206 mothers, of whom 55 were treated with metronidazole in the first trimester, 78 in the second trimester, and 84 in the third trimester. There were eight fetuses aborted or stillborn and eight babies born with developmental anomalies (3.8%). Two infants (0.9%) were considered to have major defects: one had cyanotic heart disease (mother received metronidazole treatment at 17 weeks of gestation), and the other, hydrocephaly (mother received metronidazole treatment at 35 weeks of gestation). The mother of the latter of these two infants had previously given birth to two infants with hydrocephaly.

Magnin et al.[13] reported no defects in a group of 58 infants born to metronidazole-treated mothers (three treated in the first trimester, 23 in the second, and 32 in the third).

Perl[14] reported two serious malformations, two minor skin abnormalities, and three stillbirths/deaths among infants born to 151 mothers treated with metronidazole during pregnancy (four treated in the first trimester, 77 in the second, 70 in the third). Infants were monitored for six months after delivery. One of the two malformed infants had Down's syndrome, and the other had a cardiac defect. Both mothers had been treated in the second trimester.

Pagnozzi et al.[15] treated 61 women with metronidazole for trichomoniasis during pregnancy. Three infants were born with low birth weight, but none were born with major congenital anomalies. The incidence of low birth weight was stated to be lower than usually observed.

Sands[16] treated 123 women with metronidazole during pregnancy, the majority during the second and third trimesters. One infant was stillborn, and there was one intrapartum death. The only congenital anomaly was a case of familial polydactyly. The author noted that the incidence of prematurity was 8.1%, considerably lower than the usual 11.7%.

Watt and Jennison[17] monitored 191 women treated for trichomoniasis with metronidazole; 23 of the women were pregnant. The outcome of four of the pregnancies was unknown. Sixteen normal infants were delivered, and one was born with an encephaly. Two women had abortions, one surgical.

Zacharias[18] reported that of 39 pregnant patients with adequate follow-up who were treated with metronidazole at various times between two and 8.5 months, 32 mothers delivered infants at term, and one mother delivered a preterm normal infant at 7 months of gestation. In addition, one infant with Rh incompatibility was stillborn (the mother had had two prior pregnancies that resulted in intrauterine deaths caused by erythroblastosis fetalis). There were no congenital anomalies.

Williams[19] treated 14 pregnant patients with metronidazole for trichomoniasis; no patients nor their fetuses showed any ill effects. Parker et al.[20] treated 420 patients, 36 of whom were pregnant, with metronidazole. An unstated number were treated in the first trimester. No congenital abnormalities were noted. Kotcher et al.[21] treated 36 pregnant women with metronidazole in the third trimester. Of the 34 babies for which outcome was known, one had a congenital defect: a crooked foot.

Robinson and Mirchandi[22] treated 475 women with trichomoniasis, 375 of whom were pregnant, with metronidazole. They reported on 277 women whose 281 newborns were monitored for at least six months, and most were tracked for an average of two years. This same group of mothers had previously given birth to seven infants with anatomic anomalies in pregnancies in which they had not received metronidazole. Fourteen received treatment with metronidazole was during the first trimester, 87 in the second trimester, and 176 in the third trimester. Six congenital anomalies were reported: none for women treated in the first trimester, two for women treated in the second trimester, and four for women treated in the third trimester. Eight premature births were also reported: one for a woman treated in the first trimester, four plus a second twin with toxemia to women treated in the second trimester, and two plus one stillbirth for women treated in the third trimester.

Berget and Weber[23] reviewed studies that included 1,457 infants born to 1,469 metronidazole-treated mothers, 206 of whom received the drug in the first trimester. Twenty-four children were born with malformations, three lethal, six other major, and 15 minor; there were 12 abortions and 20 deaths. The authors found incidence of malformations comparable to that of the general population and concluded that no indications could be found that metronidazole might be injurious to the fetus and its development. The studies they reviewed are included in Tables 1, 2, and 3.

Other studies, summarized in Table 1, have reported results that allowed similar conclusions to be drawn.[24–43]

Summarizing these 37 studies (which include three case reports of defects in offspring of metronidazole-treated mothers[30,40]), 5,333 mothers were treated with metronidazole during pregnancy, and 5,369 infants were born to these mothers. Two hundred eleven major and minor malformations (3.93%) were recorded. These included some infants with defects which were obviously genetic (Rh incompatibility, mental retardation in an infant born to retarded parents, familial polydactyly, Down's syndrome). One hundred eighty-nine infants (3.52%) were aborted, stillborn, or died very early in the postnatal period. Deaths included three infants from a group of seven pregnant women treated with metronidazole for amebic, pyogenic, or mixed hepatic abscess; two of the mothers died from the abscess as well.[24] If only studies including controls are considered,[5–10,22] 3,765 mothers who gave birth to 3,788 infants were treated with metronidazole; 181 infants (4.77%) had congenital defects. This compares favorably with the 10,071 defects in 166,201 control infants examined (6.06% congenital anomalies).

Both the spectrum and incidence of defects reported in infants born to metronidazole-treated mothers approximates that which one would expect in any large group of infants. In those studies that included a control group, the incidence or relative risk of defects with metronidazole treatment was similar to that found in controls.

Burtin et al.[44] have published a metaanalysis in which the safety of metronidazole in pregnancy was reviewed, using seven appropriately controlled studies.[5–7,11,12,35,40] The odds ratio for teratogenesis with vs. without metronidazole was 1.02 (95% confidence limits, 0.48 – 2.18). Odds ratio for a teratogenic effect following exposure during the first trimester was 0.93 (95% confidence limits, 0.73 – 1.18). The authors concluded that metronidazole does not appear to be associated with increased teratogenic risk.

Searle's adverse drug experience files (for which the denominator is unknown) has records of 29 reports of congenital anomalies in which metronidazole was taken at some time during pregnancy. The anomalies reported are as follows: four unspecified congenital anomalies, two hypospadias, two ectromelia, two unspecified central nervous system defects. One infant each was reported with the following defects: ventricular septal defect; spina bifida; heart defects/dislocated hip; stillbirth with malformed kidney; neuromuscular problems requiring ventilator; megakaryoblastic leukemia, hepatomegaly, splenomegaly; adrenal neuroblastoma; spontaneous abortion; malformed fetus; cerebral problems (apparently hereditary); urogenital malformation; cleft palate, ectromelia one limb; cleft palate, optic atrophy; brain damage (cord around neck; anoxia); brain damage, unspecified; encephalocele, microcephaly; facial malformation; hydrocephalus; cerebral dysgenesis, hydrocephalus, microphthalmia, seizures.

A distinct pattern of defects, such as is found with known chemical teratogens, is missing with metronidazole in both human and animal studies. For example, thalidomide caused primarily limb reduction defects, anotia, and some heart defects. Very high doses of retinoic acid have been associated mainly with facial, head, mouth, skeletal and central nervous system defects.[45] The related drugs isotretinoin and etretinate have been associated primarily with anomalies of structures that originate from the cephalic neural crest: cerebellar malformations; cerebral abnormalities; hydro- and microcephaly; mental retardation, cranial nerve deficit; skull abnormalities; ear abnormalities, including anotia and deafness; microphthalmia; facial dysmorphia; and cardiovascular abnormalities.[45,46] Tetracycline causes discolored dentition.[45,47] Streptomycin and kanamycin are associated with ototoxicity.[42] Such specificity is not evident with metronidazole.

### ANIMAL STUDIES

Controlled studies of metronidazole in rats, mice, and rabbits did not show teratogenic effects.[47] Two well-designed mouse studies[44,50] using orally dosed metronidazole found no teratogenic or fetotoxic effects; one of these was a multigeneration study.[50]

Two papers have reported both fetotoxicity and teratogenicity in rodents treated with metronidazole.[51,52] Because their results are discordant with results of other animal or human studies, a more detailed examination of these papers is warranted.

Metronidazole ("trichomonacide," stated to be identical to the French product Flagyl) was reported in a Bulgarian study to cause embryotoxic and teratogenic effects in rats, mice, and guinea pigs at dose levels corresponding to the human dose.[51] The period that the drug was administered was apparently the third trimester of pregnancy. Problems with abortions were experienced in both treated and untreated groups of guinea pigs but were more frequent in the metronidazole group. Birth defects (type unspecified) were reported in two out of five young in the treated group and three out of 26 young in the control group. In mice, "birth defects" were increased and viability somewhat decreased in the metronidazole-treated group. The rat studies were complicated by cannibalism of "a majority" of newborns in both treated and control groups. The paucity of data in the paper and the large number of deaths in both treated and control groups of all three species used make these results difficult to interpret.[51]

Giknis and Damjanov[52] reported fetotoxicity and teratogenicity in mice treated concomitantly with metronidazole and high doses of ethanol and "mild fetotoxicity" with metronidazole alone. Treatment was given on days 8, 10, 12, and 14. The route of administration of both metronidazole and ethanol used was intraperitoneal. Intraperitoneal dosing of any drug to pregnant mice presents onerous mechanical problems. In the process of four injections each in 53 pregnant mice, whose wiggling uterine horns fill the abdomen, occasional intrauterine injection is nearly impossible to avoid. That is, the actual route of administration of drug in this study may have been intrauterine in some cases, rather than intraperitoneal. The median lethal does ($LD_{50}$) of ethanol has been reported to be 7.8 g/kg.[53] The groups of mice that received ethanol were receiving 4 g/kg, more than half of an $LD_{50}$ dose, and the anesthetic effects of that much ethanol during the experimental period would likely have left these mice quite incapacitated for the better part of the day following dosing, meaning that they may not eat or even drink. Feed consumption and dam weight gain was, unfortunately, not reported.

In that study,[52] five congenital defects were reported; one in the metronidazole group, runting, is misclassified. A fetus of low birth weight would be considered small for gestational age, but low birth weight is not a malformation. Chemical teratogens, in general, produce bilateral defects, or central defects when an organ is not duplicated (e.g., ventricular septal defects) because of their uniform systemic distribution and consequent effects on specific developing structures.[45] Only two of the five malformations reported in the metronidazole group in this study–cleft palate and bilateral cataracts– fell into this category.

The statistical calculations were based on individual pups rather than litters.[52] The dams, not the pups, were individually dosed, so number of litters or dams would have been the appropriate number to use for statistical comparisons. The authors also neglected to adjust their results for multiple comparisons (Bonferroni correction); pairwise test *P*-values should be referred to 0.008 to maintain the experiment-wise error rate of 0.05. Recalculation, using the appropriate correction and group number, shows statistical significance only in the ethanol/metronidazole group. This would be expected, due to the gastrointestinal interaction between ethanol and metronidazole with consequent decrease in feed consumption. As feed consumption in the ethanol-treated groups was likely decreased anyway, this additional decrease would enhance chances of malnutrition. Given the problems with the study, their conclusions appear to be of limited or questionable significance.

### DISCUSSION AND THERAPEUTIC CONSIDERATIONS

An important consideration for use of metronidazole during pregnancy is that it has been shown to be useful in preventing premature rupture of membranes (PROM) and preterm birth, major causes of neonatal morbidity. There are comments in older literature[54] relating to the association of trichomoniasis with puerperal morbidity. Several studies have demonstrated that *Trichomonas vaginalis* infection is associated with PROM, preterm birth, and chorioamnionitis. Minkoff et al.[55,56] found that women infected with Trichomonas were significantly more likely to have PROM; effects of 15 types of microorganisms and of BV were evaluated, and Trichomonas was the only organism significantly associated with PROM. Hardy et al.[57] monitored 115 pregnant teenagers, testing for six different pathogens. Cervical Trichomonas infection, but not infection with other organisms, was significantly associated with lower birth weight and earlier delivery than in uninfected mothers or in mothers infected with other organisms; concomitant Chlamydia infection enhanced the effect of Trichomonas but did not, by itself, result in preterm birth or low birth weight.

Keith et al.[58] has shown that *Trichomonas*, a motile organism, can attach to various bacteria. They suggest that trichomonads may possibly carry pathogens through the female genital tract into the uterus and tubes and may be an important factor or cofactor in causing salpingitis that results in tubal infertility. If their conclusions are correct, Trichomonas may facilitate the ascent into the cervical canal of Chlamydia, streptococcus group B, Ureaplasma, and other bacteria known to be factors in neonatal infections, premature labor, and chorioamnionitis.

Other recent studies have indicated that development of BV during pregnancy is linked to an increased risk of preterm or low-birth-weight infants,[59–61] infection of the chorioamnionic membrane[62] or amniotic fluid,[63] and puerperal endometritis.[64,65] In a study by Morales et al.[10] women who had previously delivered a preterm infant were screened for BV between the 13th and 20th week of gestation. Eighty patients received either metronidazole, 250 mg, three times daily for seven days, or a placebo during weeks 16–20 of their pregnancy. Metronidazole, when compared with placebo, was associated with fewer hospital admissions for preterm labor, fewer episodes of PROM, and fewer infants born with a birth weight below 2,500 g ($P$<0.05). There was no significant difference between groups in the mean gestational age at delivery, although significantly fewer women in the metronidazole group delivered before 37 weeks of gestation. All women were screened for BV again at delivery. In the metronidazole group, 89% tested negative for BV, compared with 14% in the placebo group.

Hillier et al.[66] reported that in a study of the association between BV and preterm delivery of low-birth-weight infants in which more than 10,000 pregnant women were enrolled, women with BV during the second trimester of pregnancy were 40% more likely to deliver a premature, low-birth-weight infant. This relationship remained unchanged after adjustment for confounding variables. It was concluded by these investigators that prevention of even a small proportion of these births could result in large monetary savings and a decrease in neonatal morbidity and mortality.

Hauth et al.[9] studied 600 pregnant patients at risk for preterm delivery, randomly assigning them to receive one of two treatments during the second trimester: metronidazole, 250 mg, three times daily for seven days plus erythromycin base, 333 mg, three times daily for 14 days, or a placebo. These patients were screened at enrollment for BV, trichomoniasis, *Chlamydia trachomatis*, and candidiasis. Treatment was found to decrease preterm deliveries only in those patients who had BV on initial examination.

A recent publication of results of a large multicentered study (13,816 women) by Cotch et al.[67] showed that both BV and trichomoniasis at midgestation increased the risk of low birth weight and preterm delivery. Compared with uninfected women, women with both infections had a substantially higher incidence of low birth weight (13.4% vs. 6.6%), preterm delivery (17.3% vs. 10.8%), and preterm low birth weight (9.2% vs. 4.2%).

According to Hook,[68] major congenital anomalies are identified in 2–3% of liveborn infants at birth; an additional 6–7% are identified in the interval between birth and one year of age. The causes of birth defects vary; most often the etiology is unknown. Schardein[45] estimates that chemically induced birth defects probably account for only a very small fraction–about 1%–of the congenital anomalies seen in the general population. Prudence dictates that exposure during pregnancy be minimized, and that metronidazole and other drugs used during pregnancy be given at the lowest effective dose, taking into account both the benefits and the possible risk. Data collected to date do not indicate that metronidazole poses a teratogenic risk for either animals or humans.

### REFERENCES

1. Rustia M, Shubik P: Induction of lung tumors and malignant lymphomas in mice by metronidazole. J Natl Cancer Inst 48:721–729, 1972.
2. Rustia M, Shubik P: Experimental induction of hepatomas, mammary tumors, and other tumors with metronidazole in noninbred Sas:MRC(WI)BR rats. J Natl Cancer Inst 63:863–868, 1979.
3. Heisterberg L: Placental transfer of metronidazole in the first trimester of pregnancy. J Perinatol Med 12:43–45, 1984.
4. Amon I, Amon K: Placental transfer and fetal distribution of metronidazole in early human pregnancy. Int J Biol Res Pregnancy 1:61–64, 1980.
5. Rosa FW, Baum C, Shaw M: Pregnancy outcomes after first-trimester vaginitis drug therapy. Obstet Gynecol 69:751–755, 1987.
6. Morgan I: Metronidazole treatment in pregnancy. Int J Gynecol Obstet 15:501–502, 1978.
7. Heinonen OP, Slone D, Shapiro S: Birth defects and drugs in pregnancy. Littleton, MA: Publishing Sciences Group, pp. 296–303, 1977.
8. Piper JM, Mitchell DF, Ray WA: Prenatal use of metronidazole and birth defects: no association. Obstet Gynecol 82:348–352, 1993.
9. Hauth JC, Goldenberg RL, Andrews WW, et al.: Reduced incidence of preterm delivery with metronidazole and erythromycin in women with bacterial vaginosis. N Engl J Med 333:1732–1736, 1995.
10. Morales WJ, Schorr S, Albritton J: Effect of metronidazole in patients with preterm birth in preceding pregnancy and bacterial vaginosis: a placebo-controlled, double-blind study. Am J Obstet Gynecol 171:345–349, 1994.
11. Scott-Gray M: Metronidazole in obstetric practice. J Obstet Gynecol Br Comm 71:82–85, 1964.
12. Peterson WF, Stauch JE, Ryder CD: Metronidazole in pregnancy. Am J Obstel Gynecol 94:343–349, 1966.
13. Magnin P, Ambrose-Thomas P, Thoulon JM, Laurent HM: Traitement par le metronidazole de la vaginite a trichomonas de la femme enceinte. Absence d'effets teratogenes. Rev Franc Gyn Obstet 61:861–867, 1966.
14. Perl G: Metronidazole treatment of trichomoniasis in pregnancy. Obstet Gynecol 25:273–276, 1965.
15. Pagnozzi A, Pavetto PF, Malanetto C: Terapia trichomonicida in gravidanza. Minerva Gineco 21:857–860, 1969.
16. Sands RX: Pregnancy, trichomoniasis, and metronidazole. Am J Obstet Gynecol 94:35–33, 1966.
17. Watt L, Jennison RF: Metronidazole treatment of trichomoniasis in the female: Report of an extended trial. Br Med J 1:276–279, 1962.
18. Zacharias LF, Salzer RB, Gunn JC, Dierkscheide EB: Trichomoniasis and metronidazole. Am J Obstet Gynecol 86:748–752, 1963.
19. Williams BFP: Metronidazole in the treatment of trichomonal infections. Obstet Gynecol 20:611–614, 1962.
20. Parker RT, Thomas WT, Jones CP: Metronidazole in the treatment of vaginal trichomoniasis: hematologic, neurologic, and long-term clinical evaluations. Southern Med J 58:211–214, 1965.
21. Kotcher E, Frick CA, Griesel IO: The effect of metronidazole on vaginal microbiology and maternal and neonatal hematology. Am J Obstet Gynecol 88:184–189, 1964.
22. Robinson SC, Mirchandi G: *Trichomonas vaginalis.* Further observations on metronidazole (Flagyl) (including infant follow-up). Am J Obstet Gynecol 93:502–505, 1965.
23. Berget A, Weber T: Metronidazol og graviditet. Ugeskrift Laeger 134:2085–2089, 1972.
24. Nigam P, Kapoor KK, Kumar K, et al.: Hepatic abscess during pregnancy. Clinician 145–151, 1988.
25. Beard CM: Cancer after metronidazole. N Engl J Med 2:520, 1980.
26. Scott-Gray M: *Trichomonas vaginalis* in pregnancy. The results of metronidazole therapy on the mother and child. J Obstet Gynecol 8:723–729, 1961.
27. Schram M, Kleinman H: Use of metronidazole in the treatment of trichomoniasis. Am J Obstet Gynecol 83: 1284–1287, 1962.
28. Lyon FA, Sinykin MB, Maxwell MB: Trichomonal vaginitis treated with metronidazole. Am J Obstet Gynecol 85:955–958, 1963.
29. Andrews MC, Andrews WC: Systemic treatment of trichomonas vaginitis. Southern Med J 56:1214–1217, 1963.
30. Cantú JM, Garcia-Cruz D: Midline facial defect as a teratogenic effect of metronidazole. Birth Defects 18: 85–88, 1982.
31. Pereyra AJ, Lansing JR: Urogenital trichomoniasis. Treatment with metronidazole in 2002 incarcerated women. Obstet Gynecol 24:499–508, 1964.
32. Glowinski M, Chrusciel A: La trichomonase de la femme enceinte et son traitement par le 8828 R P. Gynecol Prat 15:239–246, 1964.
33. Gardner HL, Dukes CD: Clinical and laboratory effects of metronidazole. Am J Obstet Gynecol 89:990–995, 1964.
34. Whitelaw MJ, Fox LP, Schlichting FR: Results of treatment of trichomoniasis with metronidazole (Flagyl) in private and clinic patients. Western J Surg Obstet Gynecol 72:232–233, 1963.
35. Rodin P, Hass G: Metronidazole and pregnancy. Br J Vener Dis 42:210–212, 1966.
36. Csonka GW: Long-term aspect of treatment with metronidazole (Flagyl) in *Trichomonas vaginalis.* Br J Vener Dis 39:258–260, 1963.
37. Monroe SE: Trichomoniasis: clinical trial of Metronidazole (Flagyl). California Med 98:256–259, 1963.
38. Doyle JC, Rolf BB. Treatment of trichomoniasis with metronidazole. Obstet Gynecol 24:130–134, 1964.
39. Ross SM, Van Middlekoop A: Trichomonas infection in pregnancy—does it affect perinatal outcome? African Med J 63:566–567, 1983.
40. Carvajal A, Sanchez A, Hurtarte G: Metronidazole during pregnancy (letter). Int J Gynecol Obstet 48:323–324, 1995.

41. Perl C, Ragnozzi H: Flagyl in treatment of *Trichomonas vaginalis* vaginitis. Obstet Gynecol 19:595–597, 1962.
42. Robinson SC, Johnston DW: Observations on vaginal trichomoniasis in treatment with metronidazole. Can Med Assn J 85:1094–1096, 1961.
43. Doyle JC, Rolf, BB: Treatment of trichomoniasis with metronidazole. Obstet Gynecol 24:130–134, 1964.
44. Burtin P, Taddio A, Arinburnuo, et al.: Safety of metronidazole in pregnancy: A meta-analysis. Am J Obstet Gynecol 172:525–529, 1995.
45. Schardein JL: Chemically Induced Birth Defects. 2nd ed. New York: Marcel Dekkor, 1993.
46. Accutane® (Isotretinoin) detailed product information. Roche Laboratories, Inc. Nutley, NJ: vii:370–371.
47. Achromycin® (Tetracycline HCl) detailed product information. Lederle Laboratories Division, American Cyanamid Co. Pearl River, NY.
48. Bost RG: Metronidazole: toxicology and teratology. In: Finegold SM (ed.): Metronidazole Proceedings. Montreal, May 26–28, 1976. Amsterdam: International Congress Series No. 438, Excerpta Medica pp 112–118, 1977.
49. Cella PL: Ricerche sperimentali sulla teratologia del metronidazolo. Riv Patol Clin 24:529–537, 1969.
50. Chacko M, Bhide SV: Carcinogenicity, perinatal carcinogenicity and teratogenicity of low dose metronidazole (MNZ) in Swiss mice. J Cancer Res Clin Oncol 112: 135–140, 1986.
51. Ivanov I: The effect of "trichomonacide" on pregnancy in experimental animals. Akush Ginekol 8:241–244, 1969.
52. Giknis MLA, Dajanov I: The transplacental effects of ethanol and metronidazole in Swiss Webster mice. Toxicol Lett 19:37–42, 1983.
53. NIOSH Registry of toxic effects of chemical substances, 1983–84 suppl, K06300000, ethyl alcohol, p. 893.
54. Trussell R: *Trichomonas vaginalis* & Trichomoniasis. Springfield, IL: Charles C. Thomas Publishers, 1947.
55. Minkoff H: Risk factors for prematurity and premature rupture of membranes: A prospective study of vaginal flora in pregnancy. Am J Obstet Gynecol 150:965–972, 1984.
56. Minkoff H, Grunebaum AN, Schwarz RH, et al.: Prematurity: infection as an etiologic factor. Obstet Gynecol 62:137, 1983.
57. Hardy PH, Nell EE, Spence MR, et al.: Prevalence of six sexually transmitted disease agents among pregnant inner-city adolescents and pregnancy outcome. Lancet 2:333–337, 1984.
58. Keith LG, Freiberg J, Fullan N, et al.: The possible role of *Trichomonas vaginalis* as a vector for the spread of other pathogens. Int J Fertil 31:272–277, 1986.
59. Martius J, Krohn MA, Hillier SL, et al.: Relationship of vaginal *Lactobacillus* species, cervical *Chlamydia trachomatis,* and bacterial vaginosis to preterm birth. Obstet Gynecol 71:89–95, 1988.
60. Gravett MG, Nelson HP, Deroven T, et al.: Independent associations of bacterial vaginosis and *Chlamydia trachomatis* infection with adverse pregnancy outcome. JAMA 256:1899–1903, 1986.
61. Kurki T, et al.: Bacterial vaginosis in early pregnancy and pregnancy outcome. Obstet Gynecol 80:173–177, 1992.
62. Hillier SL, Martius J, Krohn MA, et al.: A case-control study of chorioamnionic infection and histologic chorioamnionitis in prematurity. N Engl J Med 319:972–978, 1988.
63. Gravett MG, Hummel D, Eschenbach DA, Holmes KK: Preterm labor associated with subclinical amniotic fluid infection and with bacterial vaginosis. Obstet Gynecol 67:229–237, 1986.
64. Watts DH, Krohn MA, Hillier SL, Eschenbach DH: Bacterial vaginosis as a risk factor for post-cesarean endometritis. Obstet Gynecol 75:52–58, 1990.
65. Rosene K, Eschenbach DA, Tompkins LS, Kenney GE, Watkins H: Polymicrobial early postpartum endometritis with facultative and anaerobic bacteria, genital mycoplasmas, and *Chlamydia trachomatis:* treatment with piperacillin or cefoxitin. J Infect Dis 153:1028–1037, 1986.
66. Hillier SL, Nugent RP, Eschenbach DA, et al.: Association between bacterial vaginosis and preterm delivery of a low-birth-weight infant. N Engl J Med 333:1737–1742, 1995.
67. Cotch MF, Pastorek JG II, Nugent RP, et al.: *Trichomonas vaginalis* associated with low birth weight and preterm delivery. Sex Trans Dis 24:353–360, 1997.
68. Hook EB: Human teratogenic and mutagenic masters in monitoring about point source of pollution. Environ Res 25:178–203, 1981.
